# Exploring self-use, attitude and interest to study complementary and alternative medicine (CAM) among final year undergraduate medical, pharmacy and nursing students in Sierra Leone: a comparative study

**DOI:** 10.1186/s12906-016-1102-4

**Published:** 2016-04-27

**Authors:** Peter Bai James, Abdulai Jawo Bah, Idrissa Momoh Kondorvoh

**Affiliations:** Department of Pharmacognosy and Phytochemistry, Faculty of Pharmaceutical Sciences, College of Medicine and Allied Health Sciences, University of Sierra Leone, Freetown First Floor Administrative Building Connaught Hospital, Freetown, Sierra Leone; Department of Pharmacology, Faculty of Basic Medical Sciences, College of Medicine and Allied Health Sciences, University of Sierra Leone, Freetown, Sierra Leone

**Keywords:** Complementary and alternative medicine, Attitude use, Pharmacy, Medical, Nursing students, Sierra Leone

## Abstract

**Background:**

CAM inclusion into the curricula of health training institutions, a strategy for its integration into the main stream healthcare delivery system is growing globally. Future healthcare professionals knowledge and perception of CAM are key determinants to its successful integration. Thus, the main objective of this study was to compare the use, attitude and interest to study CAM among final year undergraduate medical, pharmacy and nursing students at the College of Medicine and Allied Health Sciences University of Sierra Leone (COMAHS-USL).

**Methods:**

A questionnaire based cross-sectional survey was carried out among final year medical, pharmacy and nursing students enrolled at the College of Medicine and Allied Health Sciences University of Sierra Leone (COMAHS-USL). Chi square, fisher exact two tailed test and Kruskal-wallis test were used to analyze data collected.

**Results:**

Close to two-thirds (61 %) of all the three groups of final year students used one form of CAM or the other with pharmacy (72.7 %) and nursing (55.6 %) students being the highest and least users respectively. No significant difference was observed among the three groups. In general, final year students in all three cadres demonstrated a positive attitude toward CAM (33.80 ± 3. 2) with medical students showing more positive attitude than pharmacy (*p* = 0.022) and nursing student (*p* = 0.008). No significant difference in attitude was observed between students in pharmacy and nursing programs (*p* = 0.354). More than three quarter (76.6 %) of the students in all the three groups indicated their interest in studying CAM, with preference for the subject to be taught as an elective module (81.6 %).

**Conclusion:**

An appreciable number of final year medical, pharmacy and nursing students at COMAHS-USL have used at least one CAM modality and demonstrated an overall positive attitude towards CAM. Interest to study CAM was also observed among most of them even though they preferred it to be taught as an elective module.

**Electronic supplementary material:**

The online version of this article (doi:10.1186/s12906-016-1102-4) contains supplementary material, which is available to authorized users.

## Background

In recent times, the world has witnessed an increase in the use of complementary and alternative medicine (CAM) to treat diseases and also to promote health [1]. For instance, studies in the USA, Europe, and Australia have reported an increased public use of CAM [2–4]. In Africa and Asia, it is estimated that 80 % of the population use traditional medicines [1]. Also, the number of visits to a CAM practitioner and the cost incurred in the use of CAM therapies has increased considerably over the years in many parts of the world [5–7]. In Africa, one of the main CAM modalities is traditional medicine. Like most African countries, traditional medicine still remains an important source of health care in Sierra Leone [8]. A recent study conducted in Sierra Leone reported that 31 and 22 % of caregivers seek traditional therapy for diarrhea and fever for their children respectively [9].

The growing popularity of CAM has attracted the attention of policy makers, researchers and health professionals not only for its role as complementary and or alternative form of health care, but also for issues surrounding its safety and rational use especially in the African region [1]. It is a common knowledge that the quality control and safety monitoring of most of these therapies are inadequate and inefficient [1]. Notwithstanding the fact that CAM is now well established as an integral part of the main healthcare system of certain parts of the world [10]; in others, like Africa, there are growing calls for CAM to be integrated into the mainstream healthcare system [11]. In Sierra Leone, a national policy on traditional medicine was developed in recognition of the role it plays in providing primary health care. This policy seeks to provide a roadmap for traditional medicine integration into mainstream healthcare delivery system. Although its implementation still remains a challenge, key tenets of the policy include quality assurance of CAM products and training of traditional medicine practitioners and mainstream health professionals to promote rational use of traditional medicines [12].

The inclusion of CAM into the curricula of many medical, pharmacy and nursing schools has been reported in western, Asian and African countries [13–16]. Assessing the knowledge and perception of CAM among healthcare providers, including students is important to understand how well it can be incorporated into the health system and the curricula of health training institutions. Comparative studies conducted among health professionals including students, indicated a variation in knowledge, use and attitude towards CAM [17–20]. A study by Hassan and colleagues in Malaysia indicated that nursing students were knowledgeable, had a positive attitude and were willing to study about CAM than their medical counterparts [21]. Similar results were also, reported in Japan [22] and in turkey [23] In another study in Kuwait, pharmacy students were more knowledgeable in CAM than their Medical peers [24].

Literature on CAM use and attitude among health professional students in Africa are few and far between. Studies conducted in Ghana and Ethiopia showed that medical and pharmacy students generally have a positive attitude and are in support of CAM modules being included in the curriculum. The knowledge of CAM was found to be limited among Ghanaian medical students, but was found to be generally good among in Ethiopian pharmacy students [25, 26]. In Sierra Leone, pharmacy students were aware of, demonstrated positive attitude and willingness to study about CAM [27]. To our knowledge, data on comparative studies on CAM use and attitude among health professional students is limited in the African region. In Sierra Leone, no such study has been carried out. Such a study is important as it highlights variations in perceptions of health care students which do have the potential to influence the multidisciplinary delivery of health care, patient-provider relationships, and the development of training programs on CAM. It is against this background that this study was conducted to compare the use, attitude and interest to study CAM among final year undergraduate medical, pharmacy and nursing students at the College of Medicine and Allied Health Sciences University of Sierra Leone (COMAHS-USL).

## Method

### Study design and population

A quantitative cross-sectional survey was conducted among final year medicine, pharmacy and nursing students at the (COMAHS-USL) between June and August 2015. At the time of conducting this study, a total of 68 final year undergraduate students (46, 11 and 10 in medicine, pharmacy and nursing faculties respectively) were enrolled at COMAHS-USL and this formed the total study population. COMAHS-USL is the only tertiary institution in Sierra Leone that offers undergraduate degree in medicine, pharmacy and nursing. The medical and pharmacy faculties have been in existence since the inception of the college in 1988 whilst the nursing faculty was started in 2005 when the national school of nursing was incorporated into the university as a faculty. The medical, pharmacy and nursing faculties run a 6, 5 and 4 years undergraduate degree programs respectively. Based on the present curricula of all three faculties, CAM modules are only offered in pharmacy as part of the Pharmacognosy and Phytochemistry course taught at the fourth and final year. It exists as a core module in the fourth year and an elective module in the fifth year based on the subject students choose as a major to specialize on.

### Study questionnaire

The survey tool for data collection was developed based on a previously validated CAM health belief questionnaire (CHBQ) questionnaire used to asses medical student attitude towards CAM [28] with some modifications to fit our local setting (Additional file 1). The questionnaire consisted of four parts. The first part looked at student demographics such as sex, age group, program of study, place of origin, and religion. The second part attempted to assess students’ self-reported use. Students were asked to indicate whether they have used any form of CAM using “Yes or No” options. Those who answered yes were further asked to tick the CAM methods they have used. Also, they were asked to rate how effective and harmful the CAM modalities they have used are using a five point Likert scale response. The point descriptors ranged from very ineffective to very effective and from very harmful and to very not harmful. In addition, they were asked about their perceived knowledge and likelihood of recommending CAM to patients using a “Yes or No” option. Those who answered Yes were further asked to tick the CAM modalities that they will recommend and thought they are knowledgeable on. Part three sought to assess students’ attitude towards CAM using the ten item CHBQ [28] statements using a five point Likert scale response. The points descriptors ranged from strongly disagree to strongly agree. Part four evaluated respondents’ source of CAM information and their interest to study CAM using “Yes or No” options. Those who answered Yes, were further asked to tick the course options they will preferred CAM to be taught i.e. as a core or as an elective module.

### Data collection

The survey tool was distributed together with a consent form to all three groups of final year undergraduate students. To ensure compliance and higher response rate, students were asked to fill and return the questionnaires in class. They were allowed to answer the questions within 15 to 20 min. In the consent form, the essence of the study was explained and their anonymity was assured. They were also told that they have the liberty to opt out of the study at any point when filling of the questionnaire. Signing the consent form indicated they were willing to take part in the study. All filled questionnaires were collected by the second and third authors.

### Study variables measurement

Since this was a comparative study among final year undergraduate medical, pharmacy and nursing students, the independent variable was program of study whilst self-reported use, CAM effectiveness, harmfulness, recommendation and perceived knowledge of CAM were considered as dependent variables. Also, student attitude was considered a dependent variable. Each point descriptor for attitude was assigned a value. For instance, strongly disagree =1. Neutral = 3 and strongly agree =5. Attitudinal score was measured by summing up the values each respondent assigned to each of the ten statements. Thus, the minimum and maximum scores will be 5 and 50 respectively. The attitudinal score of 25 was considered as a hypothetical neutral score as was done in previous studies [27–29]. A mean attitudinal score of more than 25 was interpreted as greater endorsement and a positive attitude toward CAM by study participants. In addition, source of information and interest to study CAM were taken as dependent variables.

### Statistical analysis

Data analysis was done using SPSS Package version 16 (SPSS, Inc; Chicago). Descriptive statistics were used to calculate frequency counts and percentages for respondent characteristics and other categorical variables. In interpreting the results, Likert scale responses with a degree of agreement were grouped together as positive whilst all responses with a degree of disagreement were considered to be negative. For instance, “very effective” and “effective” were considered and grouped together as a positive response with regards to effectiveness of CAM use. To perform inferential statistics, Fisher exact two tailed test was used to compare the self reported use of CAM, recommendation of CAM, perceived knowledge of CAM, student perceived effectiveness and harmfulness and interest to study CAM as well as student sources of CAM information. The mean score for the CHBQ items that assessed respondents' attitudes were calculated for all respondents. The Kruskall Wallis test was employed to compare attitudinal scores among the three groups of final year students. The Kruskall Wallis test was used in this case because the study population was small (*N* = 64) and the data obtained for the three groups were not normally distributed. Therefore, a nonparametric equivalent of one-way ANOVA was appropriate. A pair-wise post-hoc analysis after Kruskall Wallis was also done to see which pairs of groups differ significantly. Differences were considered statistically significant if the p value was less than 0.05.

### Ethical approval

Ethical approval for this study was granted by the Research and Ethics Committee at COMAHS-USL.

## Results

### Response rate

The overall response rate for this study was 95.5 % with at least 90 % response for each cohort (Table 1).

**Table 1:** Respondents Demographics

		Medicine *N* = 46	Pharmacy *N* = 11	Nursing *N* = 10	Total *N* = 67
Response rate (n) %		44 (95.7)	11 (100.0)	9 (90.0)	64 (95.5).
Age group n (%)	21–25	15 (34.1)	2 (18.2)	2 (22.2)	19 (29.7)
	26–30	23 (52.3)	7 (63.6)	4 (44.4)	34 (53.1)
	31–35	5 (11.4)	2 (18.2)	2 (22.2)	9 (14.1)
	≥36	1 (2.2)	0 (0.0)	1 (11.1)	2 (3.1)
Sex n (%)	male	31 (70.5)	7 (63.6)	2 (22.2)	40 (62.5)
	female	13 (29.5)	4 (36.4)	7 (77.8)	24 (37.5)
Place of origin n (%)	Urban	23 (52.3)	6 (54.5)	4 (44.4)	33 (51.6)
	rural	21 (47.7)	5 (45.5)	5 (55.6)	31 (48.4)
Religion n (%)	Christian	33 (75.0)	8 (72.7)	4 (44.4)	45 (70.3)
	Muslim	11 (25.0)	3 (27.3)	5 (55.6)	19 (29.7)

#### Respondents demographics

At least half of the students in all three cohorts were within the age 26–30 years, came from urban areas and were Christians. In terms of gender, there were more males than females in medicine and pharmacy group, but not in the nursing group, although more than half of them in total were males (Table 1).

#### Pattern of use, recommendation and knowledge of CAM among final year undergraduate medicine, pharmacy and nursing students

More than half of the final year undergraduate students in medicine (59.1 %) and nursing (55.6 %) and nearly three quarter of those in pharmacy (72.7 %) indicated a previous use of one form CAM. Approximately, two thirds of medical (65.9 %) students, more than three- quarter of pharmacy students (81.8 %) and more than half of nursing students (55.6 %) indicated that they will recommend CAM to their patients. With regards to knowledge, less than half in general indicated they are knowledgeable about CAM. There was also no significant difference among the three groups with respect to self-reported use, recommendation of CAM and perceived knowledge of CAM (Table 2). Herbals/botanicals/supplements followed by spirituality/prayer were the most common modalities used across the study population. Moreover, no significant difference was observed among the three groups for all the CAM modalities considered. The same pattern was observed with respect to which modality they will recommend to patients, although in this case, a significant difference was observed with regards to herbals/botanicals/supplements. Pharmacy and nursing students were more willing to recommend herbals/botanicals/supplements than medical students. With the exception of nursing students, medical and pharmacy students indicated they were more knowledgeable about spirituality and massage than herbals/botanicals/supplements even though the difference was not statistically significant. See Table 3

**Table 2:** Comparison of the pattern of use, recommendation and knowledge of CAM among final year undergraduate medicine, pharmacy and nursing students

		Medicine n (%)	Pharmacy n (%)	Nursing n (%)	Total n (%)	*P*-value
Use of CAM	Yes	26 (59.1)	8 (72.7)	5 (55.6)	39 (61)	0.731
	No	18 (40.0)	3 (27.3)	4 (44.4)	25 (39)	
Recommendation of CAM	Yes	29 (65.9)	9 (81.8)	5 (55.6)	43 (67)	0.465
	No	15 (34.1)	2 (18.2)	4 (44.4)	21 (33)	
Knowledge of CAM	Yes	23 (52.3)	5 (45.5)	3 (33.3)	31 (48)	0.599
	No	21 (47.7)	6 (54.5)	6 (66.7)	33 (52)

**Table 3:** Comparison of the pattern of use, recommendation of and knowledge of CAM modalities among final year undergraduate medicine, pharmacy and nursing students using Fisher exact two tailed test

CAM Modalities	Medicine		Pharmacy		Nursing		*P*-value
Use of CAM							
	*N* = 26		*N* = 8		*N* = 5		
	Yes (n)	No (n)	Yes (n)	No (n)	Yes (n)	No (n)	
Acupuncture	1	25	0	8	0	5	1.000
Herbal	19	7	7	1	5	0	0.596
Massage	9	17	4	4	1	4	0.687
Ayurveda	0	26	0	8	0	5	N/A
Spiritual/prayer	15	11	5	3	3	2	1.000
Homeopathy	1	25	0	8	0	5	1.000
Meditation	4	22	2	6	0	5	0.655
Recommendation of CAM							
	*N* = 29		*N* = 9		*N* = 5		
	Yes (n)	No (n)	Yes (n)	No (n)	Yes (n)	No (n)	
Acupuncture	2	27	0	9	0	5	1.000
Herbal	14	15	7	2	5	0	0.046
Massage	11	18	6	3	0	5	0.053
Ayurveda	0	29	0	9	0	5	N/A
Spiritual/prayer	14	15	6	3	3	2	0.652
Homeopathy	0	29	0	9	0	5	N/A
Meditation	10	19	2	7	0	5	0.342
Knowledge of CAM							
	*N* = 23		*N* = 5		*N* = 3		
	Yes (n)	No (n)	Yes (n)	No (n)	Yes (n)	No (n)	
Accupunture	1	22	0	5	0	3	1.00
Herbal	10	13	3	2	3	0	0.213
Massage	8	15	4	1	0	3	0.091
Ayurveda	0	23	0	5	0	3	N/A
Spiritual/prayer	13	10	4	1	2	1	0.826
Homeopathy	0	23	0	5	0	3	N/A
Meditation	6	17	2	3	0	3	0.653

*Note*: *NA* Not applicable as no observed cases were present in the “yes” column which means Pearson chi square or Fisher exact tests could not be computed

#### CAM effectiveness, and harmfulness among final year undergraduate medicine, pharmacy and nursing students

Based on those that indicated that they have used CAM, 80 % of medical, 100 % of pharmacy and nursing students indicated the CAM modalities they used was at least effective. A similar pattern was observed with regards to the safety of the CAM modalities used. In both cases, no statistical significant difference was observed (Table 4).

**Table 4:** Comparison of CAM effectiveness and harmfulness among final year undergraduate medicine, pharmacy and nursing students using Fisher exact two tailed test

	Medicine *N* = 26	Pharmacy *N* = 8	Nursing *N* = 5	
	Effectiveness of CAM			*P*-value
Very Ineffective & ineffective n (%)	1 (3.8)	0 (0.0)	0 (0.0)	0.113
Neutral n (%)	4 (15.4)	0 (0.0)	0 (0.0)	
Effective& Very Effective n (%)	21 (80.8)	8 (100)	5 (100)	
	Harmfulness of CAM			*P*-value
Very Harmful & Harmful	1 (3.8)	0 (0.0)	0 (0.0)	0.853
Neutral	6 (23.1)	1 (12.5)	0 (0.0)	
Not harmful & Very not harmful	19 (73.1)	7 (87.5)	5 (100.0)	

#### Comparison of attitude towards CAM among final year medicine, pharmacy and nursing students

The mean attitudinal score was 33.80 ± 3. 2, which indicates a positive attitude toward CAM as it above the arbitrary mid value of 25. Analysis by Kruskal-Wallis test indicated a significant difference in attitude toward CAM among the three groups of final year students. A specific comparison testing was further done to determine specifically where differences exist between groups. Post-hoc analysis revealed that significant differences existed between medical and pharmacy students, medical and nursing students but not between pharmacy and nursing students. In both cases, final year medical students tend to have a more positive attitude toward CAM than pharmacy and nursing students (Table 5). At least 70 % of students in all three groups agreed to the statement that clinical care should integrate best conventional and CAM practices. Also, more than 80 % of student in each category believed that Health professionals should be able to advise patients on commonly used CAM methods. In addition, at least 46 % were against the notion that CAM therapies are a threat to public health (Additional file 2).

**Table 5:** Comparison of attitude towards CAM among final year medicine, pharmacy and nursing students using Kruskal-Wallis test

	Mean rank	Chi square X^2^	*p*-value
Medicine *N* = 44	37.48	10.53	0.005
Pharmacy *N* = 11	23.77		
Nursing *N* = 9	18.83		
Post-hoc Analysis			
Medicine	30.45	5.22	0.022
Pharmacy	18.18		
Pharmacy	11.59	0.859	0.354
Nursing	9.17		
Medicine	29.52	6.98	0.008
Nursing	14.67		

#### Comparison of final year undergraduate medicine, pharmacy and nursing students with regard to their interest in studying CAM

More than three quarter of all the students in the three cohorts indicated they are interested in studying CAM. Among those that were interested in studying about CAM, 81.6 % said they would prefer it to be as an elective rather than core module. A striking observation here is that all of the 90.1 % of final year pharmacy students who are interested in studying about CAM, (100 %) prefer it to be an elective module (Table 6).

**Table 6:** Comparison of final year undergraduate medicine, pharmacy and nursing students with regards to their interest in studying CAM and type of preferred module using Fisher’s exact two –tailed test

Interest in studying CAM	Medicine *N* = 44	Pharmacy *N* = 11	Nursing *N* = 9	Total *N* = 64	*P*-value
Yes n(%)	32 (72.7)	10 (90.9)	7 (77.8)	49 (76.6)	0.489
No n(%)	12 (27.3)	1 (9.1)	2 (22.2)	15 (23.4)	
If yes, type of preferred module					
Type of module	*N* = 32	*N* = 10	*N* = 7	*N* = 49	
Compulsory	7 (21.9)	0 (0.0)	2 (28.6)	9 (18.4)	0.221
Elective	25 (78.1)	10 (100.0)	5 (71.4)	40 (81.6)	

#### Sources of CAM information among final year undergraduate medicine, pharmacy and nursing students

Table 7 reveals a varying pattern with respect to sources of CAM information among the three groups of students. For medical students, the media followed by books were the highly sourced for CAM information whilst journals and books were the most CAM information sources for pharmacy and nursing students. Significant differences were observed for media and journals. Medical students access more of their information about CAM from the media than the other two groups whilst pharmacy students and nursing students sourced most of their information about CAM from journals than medical students.

**Table 7:** Comparison of the sources of CAM information among final year undergraduate medicine, pharmacy and nursing students using Fisher exact two tailed test

Source of CAM information	Medicine *N* = 44		Pharmacy *N* = 11		Nursing *N* = 9		*p*-value
	Yes	No	Yes	No	Yes	No	
Books	20	24	4	7	5	4	0.692
Media	24	20	3	8	1	8	0.027
Journals	8	36	7	4	5	4	0.002
Cam Practitioner	18	26	3	8	2	7	0.523
Other Health Professionals	2	42	1	10	0	9	0.682
Formal CAM Training	3	41	2	9	0	9	0.330

## Discussion

The main objective of this study was to compare the self-reported use, recommendation, perceived knowledge and attitude towards CAM among final year undergraduate medical, pharmacy and nursing students. In addition, their interest in studying CAM as well as their source of CAM information was evaluated. Our study recorded a higher overall response rate of 95.5 % with at least 90 % percent for each cohort. This is higher than another related study conducted in Kuwait [24] but slightly lower than the study carried out in Canada [18]. The use of an in-class data collection method is likely linked to the increased response from study participants.

Nearly, two thirds of total final year student population reported to have used one form of CAM or the other, with pharmacy students being the highest followed by medical and nursing students respectively. This is in contrast to a study carried out in the United States in which nursing students were the most CAM users [19] and in Canada in which medical students were the least users of CAM therapies [18]. The fact that pharmacy students have taken CAM modules in their pharmacognosy course at fourth year would likely have informed their decision to use CAM as compared to their medical and nursing counterparts. When comparing CAM use between medical and pharmacy students, our result is in line with a similar study conducted in Kuwait between medical and pharmacy students [24]. In all three groups, the most frequently used CAM therapy was herbal/botanicals/supplements followed by spirituality/prayer and massage respectively. This is similar to the common CAM therapies used by health science students in USA [19] and Kuwait [24] and among pharmacy students in Sierra Leone [27]. It is not surprising that herbal/botanicals/supplements were the commonly used CAM modality since it the most widely used CAM therapy in this part of the world [10]. With regards to the likelihood of recommending CAM modalities, final year pharmacy students were the most confident, followed by medical and nursing students. A similar observation was also noted by Kreitzer and colleagues [19]. It was also observed that all three groups of students will likely recommend CAM modalities that they frequently use. This would have been based on their experience of use. This hypothesis is supported by the fact that most students in all three study groups considered the CAM modalities they have used to be effective and less harmful. Similar observations were reported among health professional students in Japan [22].

Despite their likelihood of recommending CAM modalities to patients, more than half of the students in all three professions indicated that they do not have the required knowledge to properly advise patients on common CAM modalities. This might be related to students having little or no formal training on CAM in their undergraduate training. This was expected as the previous medical, pharmacy and nursing curricula used till 2014 at COMAHS-USL, were devoid of CAM modules. This, therefore, calls for the inclusion of CAM into the current curricula used especially in the medicine and nursing faculties where teaching about CAM is absent. This deficiency in knowledge might also be linked to the source of information about CAM. The result of our study indicated that final year students, especially those studying medicine, get their information about CAM from unreliable sources. As it was similarly reported in another study [18], a higher proportion of students in all three groups reported to be knowledgeable in CAM therapies that are common and frequently used in the society. For instance, medical student and pharmacy students felt they had more knowledge about spirituality/prayer as compared to nursing students who considered their knowledge on herbals/botanicals/supplements to be better than spirituality/prayer. However, a contrasting result was observed in another study in which medical and nursing students believed they are more knowledgeable in spirituality/prayer than the other CAM modalities [21]. In addition, our result highlighted differences in perceived knowledge among the three groups of final year students, although not significant. Perceived knowledge about CAM among medical students was considered to be higher than their pharmacy and nursing counterparts. This result is different from similar studies conducted in Malaysia [21] and Turkey [23] where nursing students’ perceived knowledge about CAM was higher than the other health science students.

For all three student groups, attitude towards CAM was positive and this result mirrors with other studies conducted locally [27] and elsewhere [18, 19, 21, 23–25, 30, 31]. This overall positive attitude was further demonstrated by at least 70 % of students in all three groups agreeing to the statement that clinical care should integrate the best of conventional and CAM practices. Previous experience with some of the CAM therapies might be a possible predictor of the observed positive attitude towards CAM among these three groups of students. Statistically significant difference was observed among the three cohorts of health care students. Post hoc analysis revealed that final year medical students demonstrated the highest positive attitude than pharmacy and nursing students. This is in complete contrast to studies conducted in USA [19], Japan [22] and Malaysia [21] in which nursing students had the highest positive attitude towards CAM as compared to their peers in the medical and pharmacy faculties. It is worthy to note that, in our study, no significant difference in attitude towards CAM existed between final year pharmacy and nursing students. Despite these observed differences, the overall positive attitude toward CAM demonstrated by all three categories of students, has the tendency to positively influence the way they relate to each other including CAM practitioners. Sharing the same positive mindset about CAM is also good for its integration into the mainstream healthcare delivery system.

Our study showed a limited knowledge of CAM exists among the student population. Their limited knowledge of and overall positive attitude toward CAM might be responsible for the increased interest to study CAM. This also re-echoes the need for CAM to be part of their undergraduate training. Although, no significant differences were observed among the three groups, a greater proportion of final year pharmacy students were interested in studying CAM followed by nursing and medical students respectively. This was in sharp contrast to a study conducted by Kreitzer and colleagues in which nursing students were the most interested [19]. An interesting finding from our study was that, despite their positive attitude, and interest to study CAM, most students in all three groups, preferred CAM to be taught as an elective module. A possible reason might be the fact that as final year students, they considered themselves overstretched with their major courses already and therefore consider a compulsory module on CAM to be an added burden. Although this type of teaching method has been found to be acceptable and effective among medical students [32], further studies are needed to determine whether students in lower classes will demonstrate a similar preference as this is vital when developing a comprehensive CAM instruction at COMAHS-USL. Also, future research needs to access faculty perception of CAM, all geared toward developing and implementing a CAM instruction course that will meet the needs of students in providing effective evidenced based alternative medical service to patients.

## Conclusion

Despite differences among the three groups of final year students, a higher proportion of them indicated they have used at least one form of CAM with pharmacy students being the highest and would recommend the most common CAM modalities (herbal, spiritual/prayer and massage) to their patients. However, perceived knowledge about CAM was limited with final year medical and nursing students being the highest and least respectively. Generally, all three student groups showed positive attitudes toward CAM with medical students demonstrating the most as compared to their peers. In addition, most final year students in all three groups were interested to study CAM, although they prefer it to be taught as an elective module in their respective curricula.

## Availability of data and materials

The dataset supporting the conclusion of this article is available in the [FigShare] repository, [10.6084/m9.figshare.3113281 and hyperlink to dataset in https://figshare.com/s/1aa050993380291b6d88] [33].

## Additional files

Additional file 1: Comparative study CAM survey questionnaire (DOCX 25kb) Additional file 2: Final year Medicine, Pharmacy, and Nursing students attitude towards CAM. (DOCX 14kb).
