# Supplementary material for: Exploring self-use, attitude and interest to study complementary and alternative medicine (CAM) among final year undergraduate medical, pharmacy and nursing students in Sierra Leone: a comparative study
Source: BMC Complement Altern Med. 2016 Apr 27;16:121. doi: 10.1186/s12906-016-1102-4 (PMC4847196; doi:10.1186/s12906-016-1102-4)
Supplement: Additional file 1: — Comparative study CAM survey questionnaire (DOCX 25kb) [file 12906_2016_1102_MOESM1_ESM.docx]

**Department of Pharmacognosy and Phytochemistry,**

**Faculty of Pharmaceutical Sciences**

**College of Medicine and Allied Health Sciences University of Sierra Leone.**

**Questionnaire**

**Exploring Self-Use, Attitude and Interest to Study Complementary and Alternative Medicine (CAM) Among Final Year Undergraduate Medical, Pharmacy and Nursing Students in Sierra Leone: A Comparative Study**

Dear Student,

We are conducting a ccomparative study on the self-use and attitude towards CAM among undergraduate Medical, Pharmacy and Nursing Student at College of Medicine and Allied Health Sciences university of Sierra Leone. Your input will be used to propose the development and implementation of CAM educational programs for students at COMAHS. Your responses will be anonymous and confidential and never associated with any information that could identify you personally. Only aggregated data from this survey will be reported. As there is no right or wrong answers to any item, please respond to each item according to how you feel about CAM at this point in time. Participation in this study is voluntary. You may decide not to complete this survey at any time without penalty.

Thank you.

**SECTION A: RESPONDENT DEMOGRAPHICS**

Please tick the most appropriate

**PROGRAM OF STUDY**: Medicine Pharmacy Nursing

**SEX:** Male Female

**Age Group:** 21-25yrs 26-30yrs

31-35yrs >36yrs

**Religion:** Christianity Islam

**SECTION B**

**USE OF CAM**

Have you used or currently using any form of medicine other than orthodox or conventional medicine **or western medicine**

**YES NO**

**If your answer is yes,** for each of the following 7 CAM modalities, please indicate which of them you have used or currently using , A tick in any of the circles means you are using or have used it before. **Please check all that apply.**

| CAM Modalities | **Self reported use** |
| --- | --- |
| Acupuncture |  |
| Herbal/Botanical/Supplements |  |
| Massage |  |
| Ayurveda |  |
| Spirituality/Prayer |  |
| Homeopathy |  |
| Meditation/Yoga/Relaxation |  |

If you have used any of the following above modalities, Please answer the following question

How effective do you think the modality/ies you have used is/are?

Very ineffective Ineffective Neutral

Effective Very effective

How harmful do you think the modality/ies you used is/are?

Very harmful Harmful, Neutral

Not harmful, Very not harmful

**Required knowledge and recommendation of the Use of CAM to Patients**

In your future practice as a health professional, will you recommend the use any form of CAM therapy to your patient for a particular disease condition?

YES NO

If yes, indicate which one (s) you will likely recommend to a patient. **If your answer is yes, check the box--an unchecked box indicates an answer of no. Please check all that apply.**

| Modality | **Likely to Recommend**  **to a Patient** |
| --- | --- |
| Acupuncture |  |
| Herbal/Botanical/Supplements |  |
| Massage |  |
| Ayurveda |  |
| Spirituality/Prayer |  |
| Homeopathy |  |
| Meditation/Yoga/Relaxation |  |

At this point in time, do you think you have the required knowledge to properly advise patients on the use of any form CAM?

YES NO

If yes, Please indicate which of the following CAM modalities you think you have the required knowledge to properly advise patients on the use of any form CAM? **If your answer is yes, check the box--an unchecked box indicates an answer of no. Please check all that apply.**

| modalities | **Self-reported**  **Knowledge** |
| --- | --- |
| Acupuncture |  |
| Herbal/Botanical/Supplements |  |
| Massage |  |
| Ayurveda |  |
| Spirituality/Prayer |  |
| Homeopathy |  |
| Meditation/Yoga/Relaxation |  |

**SECTION C**

**Attitude towards Complementary and Alternative Medicine (CAM)**

Please read and respond to the following statements according to your beliefs, using the numbers 1-5 **where 1 is strongly disagree and 5 is Strongly agree**.

| Statements | Strongly disagree | Disagree | Neutral | Agree | Strongly agree |
| --- | --- | --- | --- | --- | --- |
| Clinical care should integrate best conventional and CAM practice |  |  |  |  |  |
| A patient's expectations, health beliefs and values should be integrated into the patient care process |  |  |  |  |  |
| Complementary therapies include ideas and methods from which conventional medicine could benefit. |  |  |  |  |  |
| Treatments not tested in a scientifically recognized manner should be discouraged |  |  |  |  |  |
| Complementary therapies are a threat to public health. |  |  |  |  |  |
| Health and disease are a reflection of balance between positive life-enhancing forces and negative destructive forces |  |  |  |  |  |
| Effects of complementary therapies are usually the result of a placebo effect |  |  |  |  |  |
| CAM treatment have no true impact on treatment of symptoms, disease conditions |  |  |  |  |  |
| Knowledge of CAM is important to me as future a healthcare professional |  |  |  |  |  |
| Health professional should be able advise patient on commonly used CAM methods |  |  |  |  |  |

**SECTION D**

**Sources of CAM Information**

Where do you obtain information and resources for evidence-based/educational materials on CAM? (Check all that apply.)

Books Media (Radio, TV, Newspaper) Journals

CAM practitioners Other Health professionals  Formal CAM Training

Training/Apprentice with traditional medicine healer

**Interest to study CAM**

Would you be interested in learning about CAM as a module in your final year

Yes No

If **yes,** what form of CAM course would you prefer**?**

Compulsory module Elective module

***THANK YOU VERY MUCH FOR YOUR PARTICIPATION!***
