# Supplementary material for: Exploring self-use, attitude and interest to study complementary and alternative medicine (CAM) among final year undergraduate medical, pharmacy and nursing students in Sierra Leone: a comparative study
Source: BMC Complement Altern Med. 2016 Apr 27;16:121. doi: 10.1186/s12906-016-1102-4 (PMC4847196; doi:10.1186/s12906-016-1102-4)
Supplement: Additional file 2: — Final year Medicine, Pharmacy, and Nursing students attitude towards CAM. (DOCX 14kb). [file 12906_2016_1102_MOESM2_ESM.docx]

**Additional file 2 Final Year Medicine, Pharmacy and Nursing Students Attitude toward CAM**

| **statement** | **Medicine N=44** | | | | | **Pharmacy N=11** | | | | | **Nursing N=9** | | | | |
| --- | --- | --- | --- | --- | --- | --- | --- | --- | --- | --- | --- | --- | --- | --- | --- |
|  | **SD n(%)** | **D n(%)** | **N n(%)** | **A**  **n(%)** | **SA n(%)** | **SD n(%)** | **D n(%)** | **N n(%)** | **A n(%)** | **SA n(%)** | **SD n(%)** | **D n(%)** | **N**  **n(%)** | **A n(%)** | **SA n(%)** |
| ST1 | 4(9.1) | 4(9.1) | 5(11.4) | 21  (47.7) | 10(22.7) | 0(0.0) | 0(0.0) | 1(9.1) | 8(72.7) | 2(18.2) | 0(0.0) | 0(0.0) | 2(22.2) | 7(77.8) | 0(0.0) |
| ST2 | 2(4.5) | 4(9.1) | 6(13.7) | 20(45.5) | 12(27.2) | 1(9.1) | 2(18.2) | 3(27.3) | 5(45.4) | 0(0.0) | 0(0.0) | 0(0.0) | 6(66.7) | 3(33.3) | 0(0.0) |
| ST3 | 2(4.5) | 3(6.8) | 5(11.4) | 28(63.6) | 6(13.7) | 1(9.1) | 0(0.0) | 3(27.3) | 7(63.6) | 0(0.0) | 0(0.0) | 0(0.0) | 4(44.4) | 4(44.4) | 1(11.2) |
| ST4 | 5(11.4) | 7(16.0) | 10(22.7) | 12(27.2) | 10(22.7) | 1(9.1) | 5(45.4) | 2(18.2) | 2(18.2) | 1(9.1) | 2(22.2 | 3(33.4) | 4(44.4) | 0(0.0) | 0(0.0) |
| ST5 | 5(11.4) | 15(34.1) | 7(16.0) | 15(34.1) | 2(4.5) | 0(0.0) | 6(54.5) | 3(27.3) | 1(9.1) | 1(9.1) | 2(22.2 | 2(22.2 | 5(55.6) | 0(0.0) | 0(0.0) |
| ST6 | 1(2.3) | 3(6.8) | 7(16.0) | 30(68.2) | 3(6.8) | 0(0.0) | 0(0.0) | 2(18.2) | 7(63.6) | 2(18.2) | 0(0.0) | 0(0.0) | 4(44.4) | 5(55.6 | 0(0.0) |
| ST7 | 4(9.1) | 1534.1) | 16(36.4) | 9(20.5) | 0(0.0) | 3(27.3) | 2(18.2) | 5(45.4) | 1(9.1) | 0(0.0) | 0(0.0) | 1(11.1) | 8(88.9) | 0(0.0) | 0(0.0) |
| ST8 | 9(20.5) | 21(47.7) | 8(18.2) | 5(11.4) | 1(2.3) | 4(44.4) | 6(54.5) | 1(9.1) | 0(0.0) | 0(0.0) | 1(11.1) | 5(55.6) | 2(22.2) | 1(11.1) | 0(0.0) |
| ST9 | 2(4.5) | 3(6.8) | 2(4.5) | 23(52.3) | 14(31.9) | 1(9.1) | 0(0.0) | 1(9.1) | 5(45.4) | 4(36.4) | 0(0.0) | 0(0.0) | 2(22.2) | 6(66.7) | 1(11.1) |
| ST10 | 0(0.0) | 5(11.4) | 2(4.5) | 21(47.7) | 16(36.4) | 0(0.0) | 0(0.0) | 1(9.1) | 7(63.6) | 3(27.3) | 0(0.0) | 0(0.0) | 4(55.6) | 5(55.6) | 0(0.0) |

Where SD= Strongly disagree, D= Disagree, N=Neutral , A= Agree and SA= Strongly agree

Note the following:

ST1= Clinical care should integrate best conventional and CAM practices

ST2= A patient's expectations, health beliefs and values should be integrated into the patient care process

ST3= Complementary therapies include ideas and methods from which conventional medicine could benefit.

ST4= Treatments not tested in a scientifically recognized manner should be discouraged

ST5= Complementary therapies are a threat to public health.

ST6= Health and disease are a reflection of balance between positive life-enhancing forces and negative destructive forces

ST7= Effects of complementary therapies are usually the result of a placebo effect

ST8= CAM treatment have no true impact on treatment of symptoms, disease conditions

ST9= Knowledge of CAM is important to me as future a healthcare professional

ST10=Health professional should be able to advise patient on commonly used CAM methods
